# The underwood project: A virtual environment for eliciting ambiguous threat

**DOI:** 10.3758/s13428-022-02002-3

**Published:** 2022-10-26

**Authors:** Cade McCall, Guy Schofield, Darel Halgarth, Georgina Blyth, Aaron Laycock, Daniela J. Palombo

**Affiliations:** 1 Department of Psychology, University of York, York, YO10 5DD UK; 2 School of Arts and Creative Technologies, University of York, York, UK; 3 Department of Computer Science, University of York, York, UK; 4 Department of Psychology, University of British Columbia, Vancouver, Canada

**Keywords:** Uncertainty, Predictability, Anxiety, Fear, Virtual worlds

## Abstract

Threatening environments can be unpredictable in many different ways. The nature of threats, their timing, and their locations in a scene can all be uncertain, even when one is acutely aware of being at risk. Prior research demonstrates that both temporal unpredictability and spatial uncertainty of threats elicit a distinctly anxious psychological response. In the paradigm presented here, we further explore other facets of ambiguous threat via an environment in which there are no concrete threats, predictable or otherwise, but which nevertheless elicits a building sense of danger. By incorporating both psychological research and principles of emotional game design, we constructed this world and then tested its effects in three studies. In line with our goals, participants experienced the environment as creepy and unpredictable. Their subjective and physiological response to the world rose and fell in line with the presentation of ambiguously threatening ambient cues. Exploratory analyses further suggest that this ambiguously threatening experience influenced memory for the virtual world and its underlying narrative. Together the data demonstrate that naturalistic virtual worlds can effectively elicit a multifaceted experience of ambiguous threat with subjective and cognitive consequences.

**Supplementary Information:**

The online version contains supplementary material available at 10.3758/s13428-022-02002-3.

## Introduction

Danger is not always clear and present. Walking through a dark alley or swimming in opaque waters can be extremely threatening even when the exact nature of the threat is uncertain. This ambiguity necessarily shapes our response to the environment. While uncertainty can preclude taking a specific course of action, such as fighting or fleeing, one can nevertheless prepare and remain vigilant. As a chronic state, these types of responses may take the form of pathological anxiety (Grillon, 2008). But in genuinely dangerous environments, such as those confronted by first responders or military personnel, anticipating events, clarifying the nature of ambiguous threats, and preparing appropriate behaviours are adaptive responses and may even be essential for survival.

At a purely subjective level, dealing with an ambiguous threat is obviously quite distinct from confronting a concrete threat. First-hand reports of life-threatening experiences from military personnel highlight these differences (McCall & Laycock, 2022). On one hand there are ambiguously threatening, “left-of-bang” experiences in which one anticipates possible attacks while, for example, patrolling a hostile environment. In contrast are “right-of-bang” experiences during which one directly confronts a threat while, for example, being the target of enemy fire. Left-of-bang moments are characterized by feelings of apprehension, suspense, or creepiness, scanning the environment, and mentally rehearsing possible scenarios, whereas right-of-bang experiences are characterized by narrowed attentional focus on the threat, bursts of action, and an “adrenaline rush”. These subjective distinctions highlight several important features of ambiguous threat that have also been explored in the psychology and neuroscience literatures: a general state of anticipation, temporal unpredictability (i.e., when will a threat strike?), spatial uncertainty (i.e., where is the threat?), uncertainty regarding the nature of the threat (i.e., what is it?), and uncertainty regarding one’s own options for taking action (i.e., what can I do?).

Quantitative research demonstrates that, regardless of ambiguity, merely anticipating a known threat has behavioural, cognitive, and physiological consequences. Cues that a painful shock is imminent facilitate the startle reflex in both humans and rats (Bradley et al., 2005; Grillon & Baas, 2003). Such cues also elicit an orienting response in the period before the onset of the shock, as gauged by both electrophysiological and behavioural responses (Bradley et al., 2012; Nelson et al., 2015). Physiologically, this anticipatory period also elicits a decrease in heart rate (likely a consequence of the orienting response) and a concurrent increase in physiological arousal (as gauged by skin conductance; Bradley et al., 2005; Clark et al., 2012a).

Beyond mere anticipation, ambiguity itself has important consequences. One key source of ambiguity is temporal uncertainty: when (if ever) will the aversive event occur? Along these lines, threat-of-shock paradigms (e.g., Schmitz & Grillon, 2012) have revealed a great deal about the nature of ambiguous threat by comparing contexts in which shocks are delivered predictably (i.e., cued), unpredictably (i.e., at random), or not delivered at all. Unpredictable threats potentiate the startle response above and beyond predictable threats (Grillon et al., 2004, 2006). Contexts containing unpredictable threats are also subjectively more frightening, aversive, and distressing, as compared with predictable threats (Alvarez et al., 2011; Baas et al., 2004; Cornwell et al., 2008; Grillon et al., 2004). In cognitive terms, unpredictably threatening contexts elicit broadly distributed hypervigilance, whereas contexts with predictable threats elicit more focused attention to the cue or threat itself (Cornwell et al., 2008; Kastner-Dorn et al., 2018; Wieser et al., 2016). Evidence further suggests that unpredictable threats facilitate low level processing of environmental changes (Cornwell et al., 2007, 2017), biases interpretation of ambiguous cues as negative (Neta et al., 2017), and reduces risk-taking (Clark et al., 2012b). Both human and non-human animal research further demonstrates that these specific responses to unpredictable threat are subserved by distinct neural networks and neurochemical pathways (Alvarez et al., 2011; Blanchard et al., 1993; Davis et al., 2010; Kastner-Dorn et al., 2018).

While temporal unpredictability is likely to be a feature of most experiences of ambiguous threat, ambiguity has other facets. Some environments are threatening by virtue of their potential for hidden dangers. For instance, pedestrians are more likely to fear public places with dark areas or visual obstacles, ostensibly because these areas provide assailants a place to hide (Blöbaum & Hunecke, 2005; Nasar et al., 1993; Nasar & Jones, 1997). Accordingly, darkness enhances startle responses (Davis et al., 2010; Grillon et al., 1997; Jackson et al., 2003) and elevates heart rate among individuals with panic disorder as compared with healthy controls (Melzig et al., 2007). Psychopharmacological research suggests that the mechanisms underlying darkness-potentiated startle are consistent with those underlying temporally unpredictable threat (Baas et al., 2002; Davis et al., 2010). Similarly, neuroimaging research on responses to invisible versus visible threats suggests that invisible threats elicit a similar pattern of neural activity to temporally unpredictable threat (Rigoli et al., 2016).

One key consequence of threat invisibility is that it can obscure the exact nature of the threat. Imagine you are swimming at night in cloudy water and you feel something large brush against your leg. In that moment, the question is not only *if, when,* or *where*, but *what.* Less is known about the cognitive consequences of this facet of ambiguity, although anecdotal evidence would suggest that uncertainties about the exact nature of a threat are likely to trigger attempts to resolve those uncertainties. Indeed, one study found that when participants were given scenarios about potentially threatening situations, the more ambiguous the scenarios (e.g., “You are sleeping in bed during the night, but suddenly wake up thinking you have heard a suspicious noise”), the more likely they were to choose risk assessment (“check out, approach, or investigate”) as their initial defensive behaviour (Blanchard et al., 2001). Similarly, military infantry report that the experience of patrolling hostile environments is frequently pervaded with hypotheticals of who might be an enemy operative, or what object or building might hold an explosive device or trigger (McCall & Laycock, 2022).

This combination of threat with uncertainty about the source of the threat seems to elicit a relatively specific experience of “creepiness” or uncanniness (Jentsch, 1997). For example, creepy individuals are described as being both unpredictable and potentially harmful (using statements such as “I cannot predict how he or she will behave”, “I believe that he or she is intentionally hiding something from me”) (McAndrew & Koehnke, 2016). A similar pattern emerges in response to environments, where the potential of threat from uncertain sources can make spaces themselves feel creepy (McAndrew, 2020). This type of effect may even be responsible for the imagined perception of supernatural forces in allegedly haunted places. For example, some evidence suggests that people attribute sensory experiences that are difficult to pinpoint, such as a variable magnetic field, to ghosts and other creepy phenomena (Wiseman et al., 2003). So, while research in this area is limited, it seems likely that a combination of threat and uncertainty can elicit a creepy subjective response along with cognitive processing geared towards resolving uncertainty and coping with the threat.

### The virtual world

As this brief overview of the literature suggests, ambiguous threat is associated with subjective, behavioural, and physiological responses that are distinct from those that emerge when one is confronted with a concrete threat. The exploration of this distinction has been critical in furthering our understanding of the key anatomical and pharmacological differences between fear and anxiety (e.g., Cornwell et al., 2008; Grillon et al., 1991; Robinson et al., 2013; Walker et al., 2003). Indeed, experimental paradigms that create ambiguous threat via temporal unpredictability, both in animals and in humans, provide a model for examining pathological anxiety and its moderators (Charney et al., 1998; Grillon, 2008). They also provide insight into potentially adaptive responses to unpredictable and uncertain threats (Alvarez et al., 2011; Grillon et al., 2004; Neta et al., 2017) that are critical to individuals living or working in dangerous environments (e.g., first responders, military personnel, residents of hostile territory).

But existing experimental paradigms, such as those using threat-of-shock, rely on a relatively narrow scope of ambiguity by creating temporal unpredictability or by concealing known threats. Real-world threatening environments certainly include these facets of uncertainty, but they also include uncertainty regarding the nature of the threat itself and how one can best respond to it. These other facets of threat ambiguity require one to disambiguate potential signs of threat in noisy environments and to make inferences from limited information. Indeed, ambiguity regarding the nature of the threat can include temporal unpredictability and spatial uncertainty as an individual must determine if, when, and from where a threat might emerge. Moreover, complex environments open up a world of options for attention and action that are necessarily more constrained in traditional paradigms. As such, capturing these levels of uncertainty may provide further insight into the cognitive, affective, and behavioural responses to ambiguous threat.

Here we present a novel paradigm designed to elicit multiple facets of threat ambiguity via a complex and naturalistic environment. Specifically, our goal was to create a virtual world in which participants would experience uncertainty regarding the presence of threats, the nature of those possible threats, and their location in the environment. Prior work demonstrates that emotionally evocative (e.g., fearful, exciting) virtual environments (both desktop and immersive) have much to offer research on human affect (Alvarez et al., 2011; Baas et al., 2004; McCall et al., 2016; Peperkorn et al., 2015; Vermehren & Carpenter, 2020) for a variety of reasons. Experiences in virtual environments make the content inherently self-relevant by placing participants inside worlds that they can interact with and explore. These environments furthermore mimic the sensory complexity of the real world. These features enhance the ecological validity of paradigms while retaining the ability to gather subjective (McCall et al., 2015), psychophysiological (Hildebrandt et al., 2016), cognitive (Ouellet et al., 2018), and behavioural measures (Grillon et al., 2006; McCall et al., 2016) in a controlled manner.

Another feature of virtual worlds that make them ideal for studying ambiguous threat is that events can evolve over time, allowing researchers to examine dynamic responses to ongoing changes in the environment. For example, our prior work used a virtual environment to examine individual responses to a series of different stressors (e.g., explosions, vermin, a collapsing floor) that were interlaced with uneventful periods (McCall et al., 2015). Key individual differences in subjective and physiological responses to the world emerged not only in the initial reactivity to events, but also in individuals’ ability to return to calm over time and between disturbing events (Hildebrandt et al., 2016), revealing the benefits of studying participants’ responses as the environment and consequent experience unfold over time. These benefits are likely to extend to the study of ambiguous threat, particularly giventhat ambiguous threat in the real world is characterized as being more sustained than phasic (Davis et al., 2010).

To develop this virtual world, we used environmental features that have been associated with ambiguous threat in psychological research, using dark spaces (Grillon et al., 1997; Mühlberger et al., 2008) with areas where an assailant might be hiding (Nasar & Jones, 1997; Rigoli et al., 2016), limiting escape routes (Blöbaum & Hunecke, 2005; Löw et al., 2015; Nasar et al., 1993), and providing clues to the presence of a hostile agent of unknown origin (McAndrew & Koehnke, 2016).

We furthermore leveraged the expertise of horror game design to elicit the creepy feelings associated with ambiguous threat. Horror video games have been a staple of the gaming industry since its earliest days, and their history tracks innovations in using games to elicit emotion (Perron, 2009; Rouse III, 2009; Stobbart, 2019; Therrien, 2009). Here we employ some of their most effective features. These include ambient audio, which can provide players with a building sense of dread as well as cues of imminent threat or safety (Roberts, 2014), and discrete audio events that allude to unseen agent activity (i.e., footsteps) (Demarque & Lima, 2013). We used an explicit narrative to establish the potential danger of the world (Ip, 2011), one consistent with the “survival horror” sub-genre of games (Perron, 2009; Reed, 2016). We also populated the world with a variety of visual content typical of horror games and films, such as medical equipment, an overturned bed, and damaged cages (Kirkland, 2011; Steinmetz, 2018). We used lighting approaches common to horror games, with the participants seeing much of the world with a dim torchlight (Habel & Kooyman, 2014). In line with the notion that games can build narrative through their spatial architecture (Jenkins, 2003), we designed the world as a series of rooms that the participant walks through and explores. Critically, the threatening content of these rooms varies to elicit different levels of threat (Ntokos, 2018), including both ominously quiet periods and jump-scare style startling events (Baird, 2000). The order of these rooms was further designed to follow a narrative arc common across suspenseful media (Boyd et al., 2020; Lehne & Koelsch, 2015), beginning in a seemingly safe, neutral space, and then proceeding through a series of ambiguously threatening scenes, before returning to another neutral space.

### The current study

Here we tested the degree to which this virtual world succeeds in eliciting ambiguous threat. To do so, we asked participants to rate the overall experience on a variety of feeling terms. We were particularly interested in participants’ feelings of creepiness (given the putative relationship between creepiness and ambiguous threat), unpredictability (as our lay term for ambiguity), fear (as the subjective response to threat), and engagement (given that the world would have little effect if it were not engaging). If the world is indeed ambiguously threatening, then participants should rate it higher on these feeling terms (relative to others).

In addition to overall ratings of the world, we also assessed ongoing changes in subjective experience and physiological arousal. Participants provided scene-by-scene assessments of threat, unpredictability, creepiness, and anxiety. We also used these scene-by-scene variables to test our attempt to build a world with a narrative arc, beginning in a neutral state and then proceeding through a variety of ambiguously threatening circumstances, before returning to a low threat environment. Finally, we used heart rate to explore scene-by-scene threat-potentiated changes in physiological arousal and, again, to test for the influence of the narrative arc.

To test the degree to which the world could be used to test cognitive responses to ambiguous threat (proof of concept), we included two separate memory tests. First, we examined the degree to which participants’ emotional experience predicted their memory for visual features of the world. To this end, we computed measures of sensitivity and response bias. Based on the prior literature, an emotional response to the world should predict greater sensitivity for recognizing targets from the world, but bias towards perceiving targets as familiar (Bennion et al., 2013; Dougal & Rotello, 2007). These hypotheses were exploratory; however, given that the influence of emotion on memory is moderated by whether the target is central or peripheral (Levine & Edelstein, 2009), a distinction that is unclear in an ambiguously threatening environment.

Second, we tested participants’ memory for a narrative about the world, presented to them beforehand (i.e., a narrative about the damaged facilities of a sinister research project). Here the goal was to explore whether participants who were particularly affected by the world would retrospectively “confabulate” about the content of that narrative, mistakenly recognizing foils that were thematically congruent with the content of the actual world. The idea here stems from two prior findings. First, individuals with extensive experience in ambiguously threatening environments report spontaneously rehearsing their knowledge about the given threatening situation as well rehearsing hypothetical scenarios that might play out if a threat becomes concrete (McCall & Laycock, 2022). The second set of finding demonstrates that repeatedly imagining a past event can lead to an imagination inflation effect, whereby individuals falsely recognize stimuli or features of events that were imagined but not observed at encoding (Goff & Roediger, 1998; Thomas et al., 2003). Given these different findings, we reasoned that individuals experiencing a higher state of ambiguous threat by our virtual world might rehearse what they know from the narrative of the virtual world while also rehearsing potential outcomes of current events. As a combination of these processes, they might then falsely recognize thematically congruent (i.e., imagined) content, even when it was not provided by the explicit narrative.

In addition to these basic tests of the emotionally evocative nature of the world, we explored the degree to which the ambiguously threatening nature of the world is scalable via manipulating an incidental feature of the world, ambient audio. Here we expected potentially threatening music (i.e., the soundtrack from a horror film) to amplify the emotional experience of the world.

In one final sample, we explored the degree to which agency (controlling navigation in the world) is necessary to produce the world’s influence on affect, reasoning that a lack of interactivity may decrease the emotional impact of the world by decreasing the sense of presence in the environment (Welch et al., 1996)

Together, these analyses provide a comprehensive assessment of the utility of the virtual world as a testbed for examining ambiguous threat.

## Method

### Participants

Participants were recruited via a university-based system, which was open to the public. One hundred forty participants completed the study (75 women, 19 men, 1 non-binary, 45 gender not recorded; average reported age: 21; reported age range: 18 to 56). Data were gathered in three separate studies with roughly equal sample sizes (see Supplementary Table 1 for a description of each sample). These sample sizes were chosen to have sufficient power (.8) to detect within-subject changes in emotional ratings at a threshold of *p* < .05 with an effect size of *d* = .45. This effect size calculation was based on prior research which also measured within-subject changes in subjective responses to virtual environments (McCall et al., 2016). To acquire enough data to reach the suggested sample of at least 40, we recruited approximately 50 participants per sample to accommodate for drop-out. Common measures were used across these three studies (see Supplementary Table 1 for a breakdown of measures per sample). For each analysis, we used all participants who had completed the relevant measures.

### Materials

#### The underwood project (the virtual world)

The Underwood Project is a modular virtual environment kit built in the Unity game creation environment with standard packages (available here: https://drive.google.com/drive/folders/1BAk2eL1sfD1OemQmEclQ5FoJ0N_yI1AT?usp=sharing). The version of the world used in the studies present here was built in Unity 5.5 (although the publicly available kit has been updated to Unity 2021.1.26f1). The 3D models within the kit were developed in 3ds Max 2017. The Underwood Project is designed in a modular fashion in order to enable individuals with little experience of Unity to make relatively complex interactive environments.

Participants experienced the world through a three-monitor set of displays and navigated via a handheld controller. The displays (23″ diagonal LED) were arranged horizontally with the side displays oriented in at approximately 30 degrees. This arrangement was used to increase the field of view and, as a consequence, increase presence (Cummings & Bailenson, 2016). In the version of the world used here (executable version available on the OSF repository), the participant proceeds through a series of rooms, each with its own unique features. Within each room, the participant must find the key for the door to the next room. Participants pick up items (e.g., keys, a torch) or press buttons, by getting close to them. Upon picking up the key in a given room, the next door opens.

##### Music manipulation

In one subsample (see Supplementary Table 1), we tested the degree to which horror-associated music could scale the world’s effects. This music was either music from a horror film (Carlos & Elkind, 1980) or relatively neutral music (Enea, 2006).

##### Interactivity manipulation

In another subsample (see Supplementary Table 1), we manipulated whether or not participants had control over the viewpoint. Participants in the interactive condition navigated through the world as with the previous studies. Participants in the passive condition passively watched a recording of the experience from a past participant; each participant in the passive condition was yoked to one of the participants in the interactive condition.

#### Questionnaires

##### Overall affect

We assessed participants’ overall affective response to the world with a questionnaire. Participants used slider scales ranging from “not at all” (0) to “a moderate amount” (50) to “a great deal” (100) to rate the degree to which the world was “frightening”, “creepy”, “unpredictable”, “amusing”, “funny”, “engaging”, “confusing”, “disgusting”, “interesting”, “surprising”, “frustrating”, “sad”, “boring”, and “enjoyable”. We chose the first three of these words because they are particularly relevant to ambiguous threat and thus serve as a manipulation check. We chose the other words because they are commonly used in research on emotional responses to media (Gabert-Quillen et al., 2015; Gross & Levenson, 1995; Rottenberg et al., 2007). The order of the words was randomized between participants.

##### Scene-by-scene affect

We assessed participant’s scene-by-scene subjective responses with an additional questionnaire. After completing the task, participants watched eighteen short videos of different rooms in the world. These rooms were presented in the order in which they appeared in the virtual world. In the first study and the study on ambient music, participants were asked to report the degree to which they found the room to be “creepy”, “unpredictable”, and “threatening” on a sliding scale from “not at all” to “very much so”. These words were chosen to test the notion that perceived creepiness is a combination of threat and uncertainty. In the interactivity study, participants were asked to rate each room on the degree to which it was anxiety-provoking and unpredictable. These words were chosen to test the possibility that being under threat without control over movement would amplify unpredictability and anxiety.

##### Scene memory

After completing the task in the virtual environment, participants were presented with a series of 48 randomly presented still images. Half of these were from the environment that the participants saw, and the other half were foils created from different environments or including objects that were not present in the test environment. Participants were asked to report whether they recognized each image from the virtual world. We used these data to calculate a hit rate and a false alarm rate. We also used the Psycho package (Makowski, 2018) for R to calculate *d*-prime (*d*′) as a measure of sensitivity and *c* as a measure of bias (where a lower score reflects a more liberal criterion for responding “yes”). When calculating *d*′, we used the adjustment for values (e.g., a hit rate of 1 or a false alarm rate of 0) recommended by (Hautus, 1995), as implemented in the Psycho package.

##### Prelude memory

Before beginning their task in the virtual world, participants listened to a prelude which described the content of the virtual world. This prelude explained that the participant was about to enter The Underwood Labs. It suggested that something had gone wrong in the labs but was not clear about the nature of the problem. After the task was completed, participants were provided with a list of thirty randomly presented statements and asked which statements had been made in the prelude. One third of these statements were actual statements from the prelude, another third were foils that were thematically congruent with threat (e.g., “Screams emerged from the building yesterday”) and a final third were foils that were not (e.g., “The laboratory employs many locals”). From these data, we calculated a hit rate, a false alarm rate, *d*′ and *c* using the same methods as the scene memory variable. In addition, we calculated a difference score to assess thematically congruent confabulation by subtracting the false alarm rate for neutral foils from the false alarm rate for affectively congruent foils.

### Heart rate

In addition to subjective and cognitive measures, we also measured heart rate over the course of the world. We recorded ECG using a AcqKnowledge 5.0 software (Biopac Systems Inc., Santa Barbara, CA), a Biopac MP160 acquisition system, and a Biopac BioNomadix electrocardiography (BN-RSPEC) amplifier with a three-lead set of pregelled Ag/AGCl foam electrodes. Electrodes were placed on the sternal end of the right clavicle, the left mid-clavicle (grounding), and the lower left rib cage. Data were recorded at 2000 Hz. Event related timestamps were recorded on the rendering computer and onset time of physio acquisition was recorded on the physio acquisition computer (along with sample-by-sample timestamps). The system clocks on both computers were synched, which allowed us to align these data series.

In terms of preprocessing, R peaks were identified using AcqKnowledge software. We visually inspected R-R tachograms to identify and remove extra R peaks and to add missing peaks (i.e., to label R peaks that fell below the algorithm’s threshold). For five participants, there were too many artefacts and were removed from analysis. R peak timing was then exported from AcqKnowledge for analysis. We calculated instantaneous HR using the RHRV 4.6 package in R based. We then aggregated data per scene to produce an HR value for each scene references in the scene-by-scene affect data. HR was calculated for samples A and B, the two samples for which we had gathered per-scene timing data.

### Procedure

Participants first completed a questionnaire covering basic demographic information. They were then hooked up to the physiological equipment. Next, the participant listened to the prelude. After that, an experimenter provided the instructions for the virtual world: “When we start the virtual world, your only task will be to walk through the world. You will frequently encounter locked doors. To open a door, find the key in the room. To pick up a key or any other object, just walk towards it. At a couple of points, you will also need to push an elevator button. Again, just move towards the button in order to press it.” The experimenter then started the world. Once the participant confirmed that they could hear the audio though the headphones and that they could navigate using the controller, the experimenter asked the participant to proceed with the task.

We designed the sequence of rooms in this instantiation of the world to go from a relatively neutral space (entrance in Figs. 2 and 3) to a series of ambiguous threatening spaces, before returning to another relatively neutral space (exit). To limit escape routes (Blöbaum & Hunecke, 2005; Löw et al., 2015; Nasar et al., 1993), a freight elevator takes participants down to a subterranean level (elevator down) which can only be accessed via the elevator. To leverage the ambiguously threatening potential of darkness (Grillon et al., 1997; Mühlberger et al., 2008), the lights on the subterranean level are out and the participant can only see via a torch which projects light from their viewpoint to the space immediately in front of them (from dark basement until long bright hallway). To amplify ambiguous threat by providing areas where an assailant might hide (Nasar & Jones, 1997; Rigoli et al., 2016), the subsequent rooms include dark corners, shelves, boxes, and clouds of steam (e.g., dark office, storage room, flares and steam, shadow). Some rooms further provide clues that an unknown and potentially hostile agent might be present or nearby (McAndrew & Koehnke, 2016) via the sound of footsteps (footsteps), scattered bones and blood stains (blood and bones), a shadow of a moving figure (shadow). The world also includes startling events (Baird, 2000), one in which a door slams abruptly (door slams) and one in which an underground train rides past (train). The threatening nature of the world culminates by combining the different sources of ambiguous threat (something approaches). Participants enter an elevator. After pressing the button, they must wait for the elevator door to close. While waiting, the lights begin to go out in the hallway in front of the elevator. As the darkness gets closer, a screeching sound and footsteps get louder, as though something is approaching in the shadows. At the end of the experience, the participant takes an elevator up to a brightly lit upper level (narrow escape, back upstairs and exit). On average, navigating through the entire world lasted approximately 10 minutes (M = 9.7, SD = 3.6).

Immediately after participants had completed the task in the virtual world, they completed the prelude memory test, scene memory test, scene-by-scene affect questions, and overall affect questions (in that order).

### Analyses

For all variables, we identified outliers using the robust median absolute deviation method as implemented in Routliers R package (Delacre & Klein, 2019; Leys et al., 2019). For each outlier identified, we confirmed that the value was within a feasible range for the variable. We also reran all analyses using the given variable but omitting the outlier values. Here we report the outcome of all analyses (with and without outliers).

All linear mixed models (LMMs) were fitted in R 4.0.5 (64) using the lme4 1.1-26 package (Bates et al., 2015). We obtained *p*-values for *F* and *t*-tests using the lmerTest package 3.1-3 (Kuznetsova et al., 2017) ANOVA function using Satterthwaite’s method. We calculated estimated marginal means (with 95% confidence intervals) using the emmeans package 1.6.3 (Lenth, n.d.). Post hoc pairwise comparisons were also calculated using the emmeans package with a Tukey correction for *p*-values.

For a partial least squares (PLS) analysis, we used the PLS command line package (Version 6.15, 2015) in MATLAB R2021a. PLS is a technique that models data in terms of latent variables (LVs) in order to explain maximal co-variance among matrices of *X* and *Y* variables (see Krishnan et al., 2011 for details). In our analysis, the “*X*” variables consisted of emotional responses and the “*Y*” variables consisted of memory measures. Values in both matrices were first scaled, namely transformed into *z*-scores, before inputting to the PLS analysis. In brief, PLS uses singular value decomposition (SVD) to construct covariance matrices between the inputted *X* and *Y* variables, which are then decomposed into orthogonal vectors of singular values. Permutation testing was conducted to test the overall significance of the LVs (i.e., randomizing the order of observations from the *X* matrix 1500 times [*Y* is left unchanged] and performing SVD on each permutation to obtain singular values to derive *p*-values). LVs with *p <* .05 were considered statistically significant. Bootstrap resampling was conducted such that observations from both the *X* and *Y* matrices were sampled (with replacement) 1000 times. This approach yields bootstrap ratios (BSRs). BSRs are conceptually analogous to *z*-scores in that bootstrap ratios exceeding ±1.96 represent significant correlations. With respect to interpreting the specific pattern of results produced by the PLS analysis, a memory variable was considered a significant contributor to an LV if its correlation coefficient was significantly different than zero (i.e., if the 95% CI did not cross the zero line). An emotion variable was considered a reliable contributor to an LV if its BSR exceeded a value of ±1.96.

All data, the study materials, and the supplementary materials (SM) are available in the OSF repository, https://osf.io/euvgt/?view_only=d76a74fc86ba40408bebe9b1b1e29ecf.

## Results

### Overall subjective experience

**Fig. 1:**
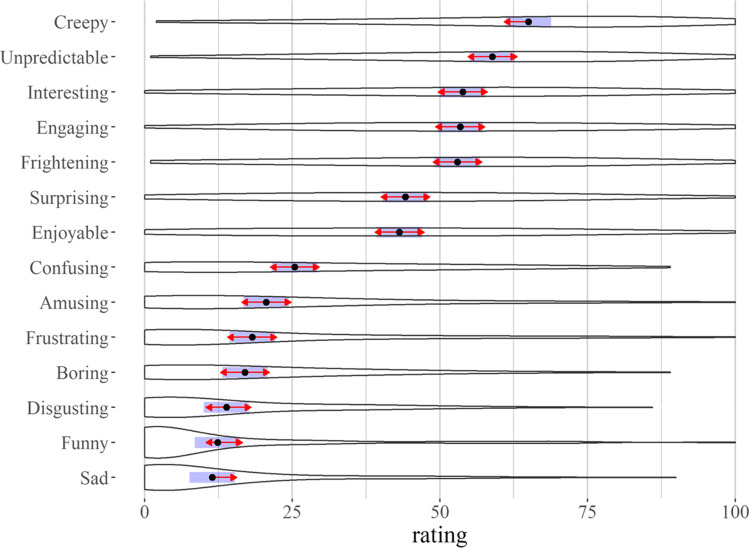
Ratings of overall subjective experience. The estimated marginal means (black dots), their confidence intervals (purple line), and distributions (violin plots) for ratings of the overall experience of the virtual world. The red arrows are for comparisons between means. Overlapping arrows between two means indicates that the difference is not significant based on the adjusted *p*-values

We used a LMM to examine the relationships between ratings of the different words participants used to report their overall feelings about the world. This model predicted the rating level with a fixed effect for feeling word (e.g., “creepy”) and a random effect for participant intercept. The feeling with the highest average rating, “creepy”, was used as the reference level. A significant effect of feeling word emerged (*F*(13, 1807) = 127.04, *p* < .001). Parameter estimates and confidence intervals are reported in supplementary materials (SM, “Overall Affect: Model Summary”). Pairwise comparisons of the estimated marginal means revealed significant contrasts (SM, “Overall Affect: Pairwise contrasts”). Figure 1 illustrates the estimated marginal means and confidence intervals. The red arrows in Fig. 1 illustrate the post hoc contrasts between means; overlap between any pair of red lines indicates an absence of a significant difference for that contrast. “Creepy” (M = 65.0, CI = [61.2, 68.9]) was rated significantly higher than all emotions except “unpredictable” (M = 58.9, CI = [55.0, 62.8]), and the mean for bothfell between “a moderate amount” and “a great deal” on the scale. There were no other significant differences between the top five rated feelings which, in addition to “creepy” and “unpredictable”, included “interesting” (M = 53.9, CI = [50.0,57.7]), “engaging” (M = 53.4, CI = [49.6, 57.3]), and “frightening” (M = 53.0, CI = [49.1, 56.8]). “Surprising” (M = 44.2, CI = [40.3, 48.0]) and “enjoyable” (M = 43.1, CI = [39.3, 47.0]) were rated significantly lower than these five, but higher than the remaining reported feelings. The lowest-rated words (“amusing”, “funny”, “disgusting”, “frustrating”, “sad”, and “boring”) had a floor effect with extreme positive skew (estimated skewness > 1; Joanes & Gill, 1998). Given the potential for these skewed distributions to bias the LMM, we ran a second model excluding the skewed variables. The reported pattern of results among the remaining variables held (SM). Together these data demonstrate that the virtual world successfully elicited subjective feelings associated with ambiguous threat—creepiness, unpredictability, and fear—as well as general engagement.

### Scene-by-scene affect

**Fig. 2:**
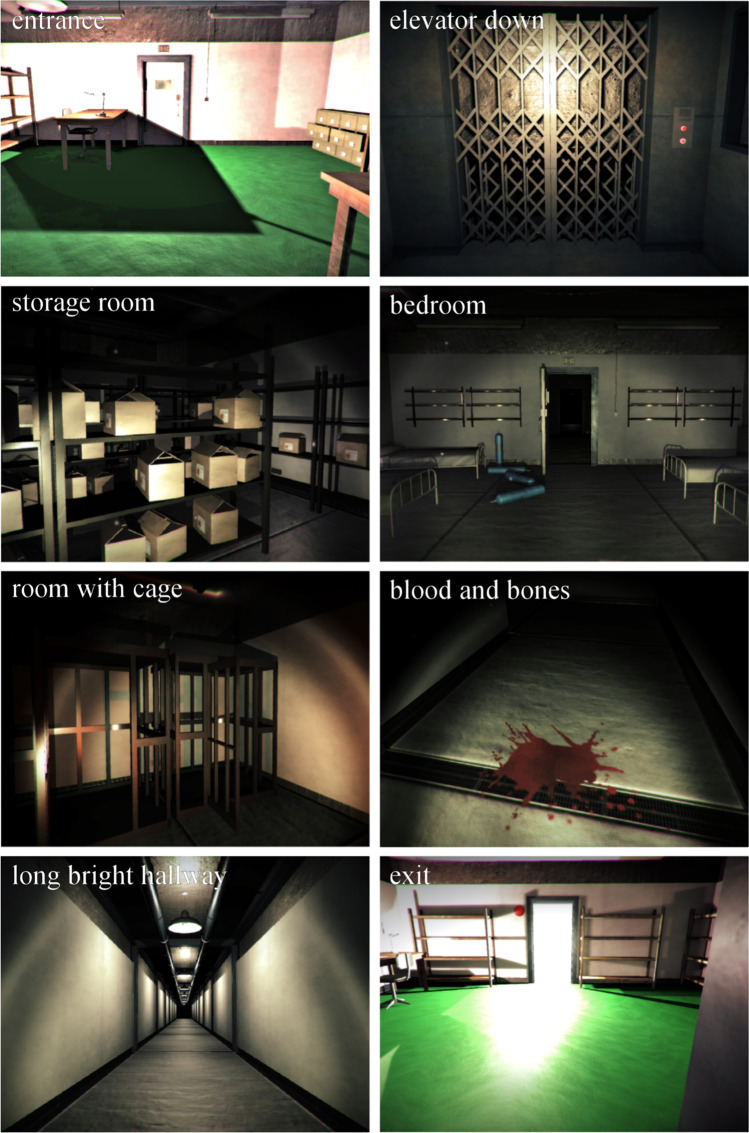
Screenshots from the virtual world. These are a subset of the scenes in Fig. 3

**Fig. 3:**
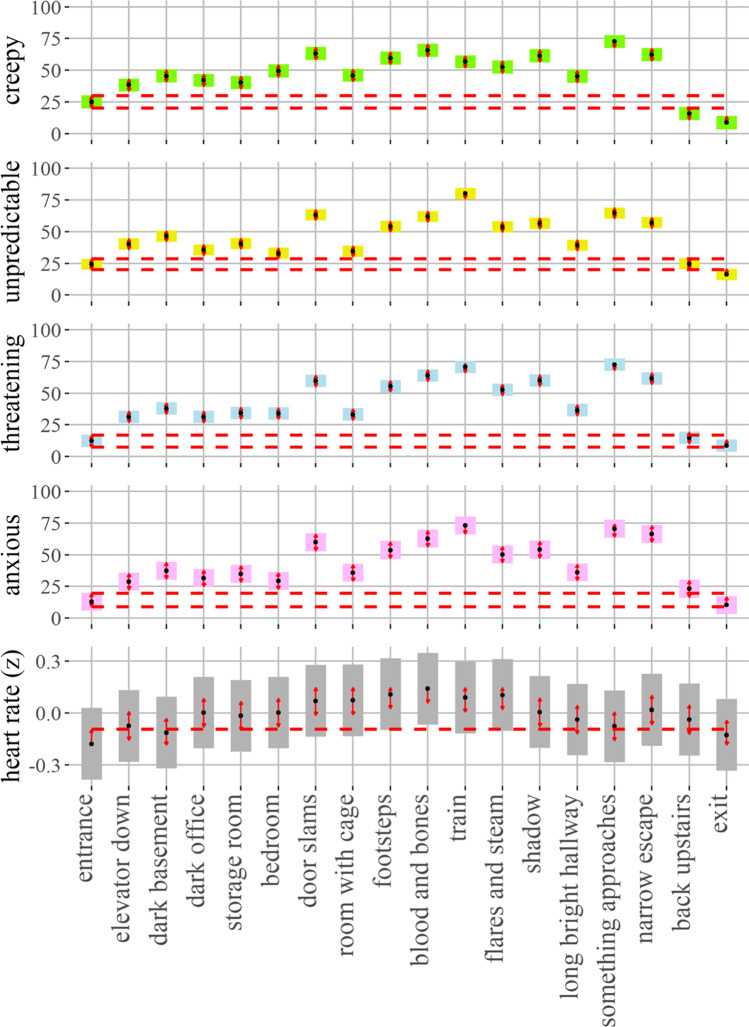
Scene-by-scene affect and heart rate. The estimated marginal means (black dots) and their confidence intervals (shaded bars) for the scene-by-scene ratings and heart rate levels. The red arrows are for comparisons between means. Overlapping arrows between two means indicates that the difference is not significant based on the adjusted *p*-values. The order of the scenes (*x* axis) is chronological

We used LMMs to test scene-by-scene levels of creepiness, threat, predictability, and anxiousness (see Fig. 2 for sample images of scenes). Each of these models predicted rating for the given term using a fixed effect for scene and a random effect for participant intercept. Scene had a significant effect for all models, predicting changes in creepiness (*F*(17, 1640) = 77.0, *p* < .001), threat (*F*(17, 1640) = 119.0, *p* < .001), unpredictability (*F*(17, 2354) = 95.6, *p* < .001), and anxiety (*F*(17, 697) = 53.8, *p* < .001). Estimated marginal means, confidence intervals, and pairwise contrasts are illustrated in Fig. 3. Within that figure, scenes are presented in chronological order, demonstrating that participants experienced an arc of emotional experience that waxed and waned for all four scene-by-scene variables (tests of pairwise comparisons between scenes are illustrated with the red arrows). Pairwise comparisons between each scene and the entrance scene reveal that all scenes were rated higher than the original scene on all variables, except for the final two scenes (*back upstairs* and *exit*); this pattern of contrasts held for creepiness (all *t*.ratios (1640) > 4.8, all *p*s < .001), threat (all *t*.ratios(1640) > 7.3, all *p*s < .001), unpredictability (all *t*.ratios(1640) > 3.6, all *p*s < .01) and anxiousness (all *t*.ratios(1640) > 4.3, all *p*s < .001). Model summaries, estimated marginal means, and pairwise contrasts are provided in the SM. These analyses demonstrate that the world elicited our desired arc of subjective experience, building ambiguous threat from a relatively neutral state before returning to neutral at the end.

We ran a number of additional analyses to examine the relationship between scene-by-scene reports of creepiness with the overall creepy evaluation of the virtual world. We correlated ratings for each scene with the overall rating of creepiness, adjusting for family-wise errors via the Holm method (Holm, 1979) in the psych package of R (Revelle, 2021). All correlations were significant (all *r*s > .23, all *p*s < .03, all *N*s = 98). Correlations with overall creepiness were lowest at the beginning (*entrance*, *r*(97) = .25, *p* = .03) and end (*exit*, *r*(97) = .23, *p* = .03) of the world and peaked in the middle (*flairs and steam*, *r*(97) = .65, *p* < .001), although all confidence intervals overlapped. The SM includes a table of those correlations. These analyses suggest that participants’ overall evaluations of the affective experience of the world were not driven by either primacy or recency effects, but mainly by the central content of the world.

### Scene-by-scene heart rate

We used another LMM to examine changes in heart rate over the arc of the experience. This model predicted heart rate (*z*-scored across participants) using a fixed effect for Scene and a random effect for participant intercept. Scene had a significant effect (*F*(17, 1531) = 7.03, *p* < .001). Estimated marginal means, confidence intervals, and pairwise contrasts are also illustrated in Fig. 3. Pairwise comparisons between each scene and the entrance scene reveal that heart rate rose above its level in the entrance room from the *dark office* (*t*.ratio(1531) = 3.8, *p* = .002) through the *long bright hallway* (*t*.ratio(1531) = 2.9, *p* = .043), peaking in the middle of the world (*blood and bones*, *t*.ratio(1531) = 6.7, *p* < .001). Heart rate rose again during the *narrow escape* (*t*.ratio(1531) = 4.1, *p* < .001) variables, before returning to levels similar to the *entrance* in the final two scenes, *back upstairs* (*t*.ratio(1531) = 2.9, *p* = .05) and *exit* (*t*.ratio(1531) = 1.1, *p* = .92). Several samples for two participants exceeded the upper limit of our outlier threshold, although the heart rates were still well within the healthy range. We reran the above analyses omitting those samples. The pattern of results remained the same. Model summaries (with and without outliers), estimated marginal means, and pairwise contrasts are provided in the SM.

To examine the relationship between scene-by-scene subjective affect and heart rate, we ran an additional mixed model. This model predicted heart rate in each scene with fixed effect terms for per-scene ratings of threat, creepiness, and unpredictability (all of the scene-by-scene affect terms we had for participants with heart rate data) and random effects for scene intercept and participant intercept. Threat (*F*(1,1141.2) = 6.61, *p =* .01), but not creepiness nor unpredictability (*p*s > .05), had a significant effect with higher levels of subjective threat predicting higher heart rates. Again, this pattern remained the same when outliers are removed.

These analyses suggest that physiological arousal mapped onto the affective arc of world and was specifically associated with the experience of threat.

### Affect and memory

As an initial analysis of the memory data, we first examined participant performance. Participant *d*′ scores for scene memory were significantly greater than 0 (M = 1.45, SD = .36, *t*(137) = 47.0, *p* < .001). Similarly, *d*′ scores on the prelude memory task were significantly greater than 0 (M = .99, SD = .57, *t*(139) = 20.8, *p* < .001).Together these data suggest that participants were performing above chance on the memory measures. Scores on our measure of contextually congruent confabulation about the prelude test (congruent false alarm minus incongruent false alarm rate) (M = .31, SD = .20) were significantly greater than 0 (*t*(139) = 18.1, *p* < .001), demonstrating that participants were more likely to falsely recognize the thematically congruent foils.

**Fig. 4:**
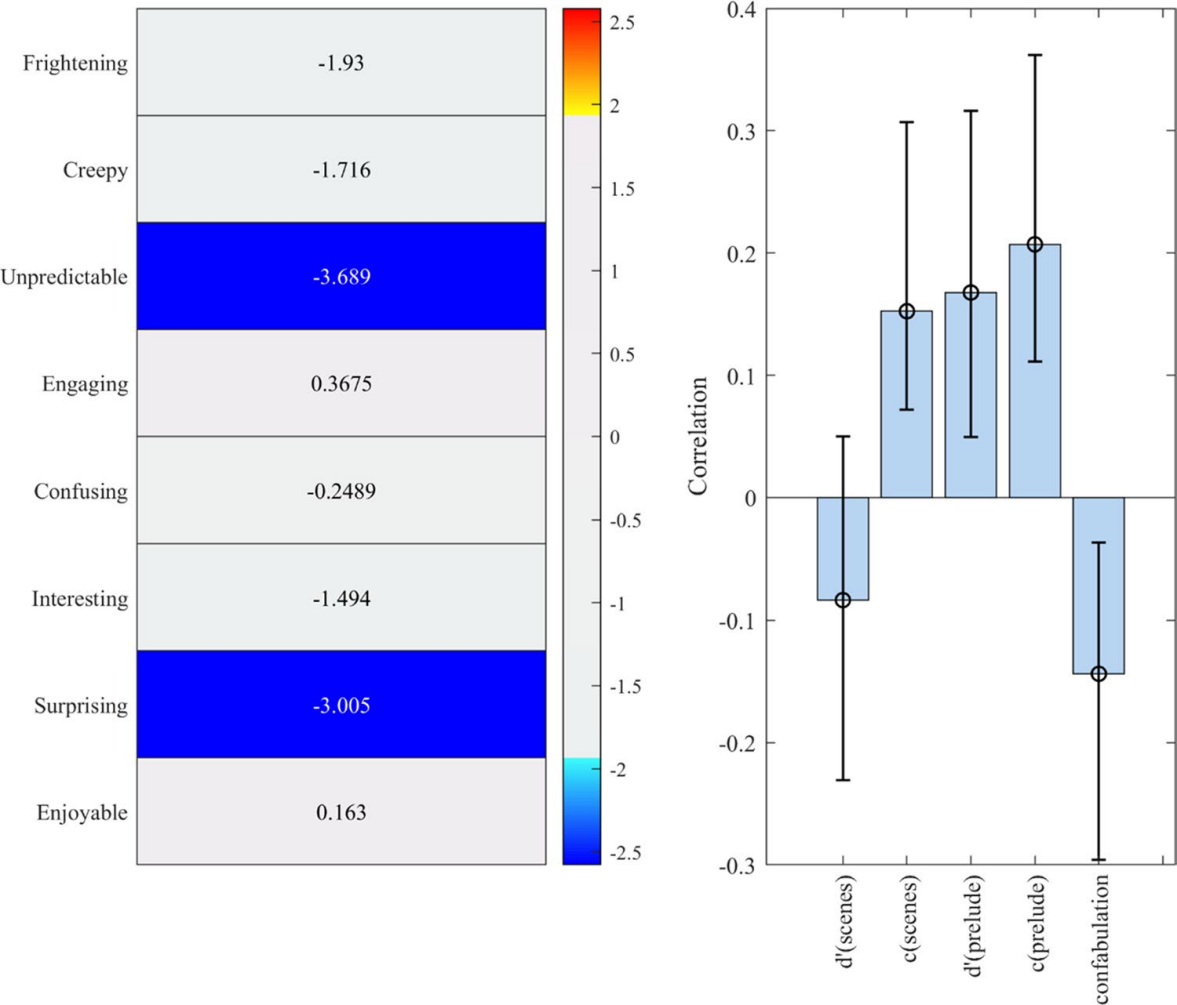
Relationship between affect and memory. One latent variable (LV) emerged (*p* < .01) in the partial least squares (PLS) analysis demonstrating an association between memory measures and overall emotions. Ratings of unpredictability and surprise contributed to the LV, which correlated with memory bias for both the scenes and the prelude, and with sensitivity (*d*′) and “confabulation” about the prelude (see main text)

To test the relationship between affective responses to the world and memory, we ran a partial least squares model (see Fig. 4). One significant LV emerged (*p* = .01). Together this pattern suggests that the more unpredictable and surprising individuals found the world, the weaker their sensitivity (*d*′) in the prelude memory task, the more liberal their criteria for recognition in both the scene and prelude memory task (*c*), and the more likely they were to false alarm on thematically congruent, but not incongruent, statements from the prelude. This pattern of results remained the same when we reran the analysis after omitting participants whose memory variables exceeded our outlier threshold (4 participants), although “frightening” joined “unpredictable” and “surprising” as significant contributors to the pattern (see SM). Thus, these data suggest that the more participants found the world unpredictable, the more likely they were to falsely recognize its content and the narrative, particularly with reference to thematically congruent content.

### Overall affect and ambient music

To test the effect of ambient music on overall subjective experience, we adapted the linear mixed model used above in the overall subjective experience analysis. This model predicted emotion ratings from a fixed effect of music condition (neutral versus horror) and emotion type. The model also included a random effect for participant intercept. Significant effects of emotion type (*F*(13, 611) = 45.2, *p* < .001), condition (*F*(1, 47) = 9.76, *p* = .003) and their interaction (*F*(13, 611) = 2.11, *p* = .01) emerged. Unexpectedly, the neutral music condition (M = 40, 95% CI [35, 43]) elicited stronger emotional responses than the horror music condition (M = 30, 95% CI [26, 35]). To probe the interaction effect, we ran post-hoc comparisons between conditions for each emotion type. The neutral music condition was rated as significantly more unpredictable, engaging, interesting, and surprising. However, the between condition contrast for unpredictability was the only one to survive a Bonferroni correction (M = 28, ***t***.ratio (470) = 2.78, *p* < .001).

### Overall affect and interactivity

To test the effect of interactivity on overall subjective experience, we again adapted the linear mixed model used above in the overall subjective experience analysis. This model predicted emotion ratings from a fixed effect of interactivity condition (interactive vs passive) and emotion type. The model again included a random effect for participant intercept. Significant effects of emotion type (*F*(13, 520) = 44.6, *p* < .001), no effect of condition (***F***(1, 40) = 3.06, ***p*** = .09), but an interaction between emotion type and condition (*F*(13, 520) = 5.4, *p* < .001) emerged. To probe the interaction effect, we ran post-hoc comparisons between conditions for each emotion type. The interactive condition was rated as significantly more unpredictable, engaging, interesting, surprising, and enjoyable and less frustrating. However, the between condition contrast for finding the world interesting was the only one to survive a Bonferroni correction (M = 34, ***t***.ratio (296) = 4.7, *p* < .001).

## Discussion

In the work presented here, we designed and tested a virtual world for eliciting ambiguous threat. To determine the content of the world, we drew upon evidence from psychology and neuroscience, as well as expertise from video game design. In line with our goals, participants found the world creepy above all else. Scene-by-scene reports of creepiness revealed that this feeling emerged once the participants left the world’s initial, safe space and persisted until the participants returned to a final, safe space. Participants reported a similar narrative arc in their feelings of unpredictability, threat, and anxiety. Physiological arousal, as gauged by heart rate, also peaked in the middle of the world and correlated with individuals’ experiences of threat.

Additional analyses illustrate the world’s potential for studying the effects of ambiguous threat on cognition. We used two memory tests, one to probe memory for the world’s content and one to probe confabulation about the explicit narrative of the world. For both of these measures, participants’ subjective response to the world predicted memory bias. This finding is generally consistent with some simpler laboratory studies of emotional memory (Bennion et al., 2013; Dougal & Rotello, 2007), wherein participants study emotional images, such as a car crash or a mutilated face, or emotional words. But these types of memory phenomena have yet to be assessed in the context of ambiguous threat. This difference might be critical, as in the present study, the experience of ambiguity itself seems key in influencing memory. Participants who found the world unpredictable and surprising displayed a bias towards believing they had seen target stimuli. Unlike some prior work, however, we did not find a relationship between emotion and sensitivity (i.e., *d*-prime) in terms of memory for the scenes, as our results were specific to indices of bias. One important point to note here is that the current paradigm is difficult to frame in terms of the existing research on differential effects of emotion on memory for central versus peripheral events (Levine & Edelstein, 2009); stimuli in the current study were peripheral in the sense that they were not the source of a threat, but the term “peripheral” may be inappropriate in contexts such as this one where there was no central, concrete source of threat. Regardless, the pattern of subjective uncertainty was also associated with reduced sensitivity with respect to recognition of the prelude narrative and with false alarming on thematically congruent (but not incongruent) foils from the world’s prelude voiceover. This latter effect suggests that ambiguously threatening environments might encourage processes such as rehearsal of hypothetical scenarios (McCall & Laycock, 2022) which may, in turn, lead to memory interference between real and imagined events (Goff & Roediger, 1998; Thomas et al., 2003). While these exploratory findings require follow-up to replicate and probe, they illustrate that this virtual world is a potentially useful tool for understanding the cognitive effects of experiencing ambiguous threat in environments that are, like the real world, complex, dynamic, and interactive. Critically, such a paradigm could be used to interrogate the mechanisms for mnemonic effects in a naturalistic context by, for example, measuring eye movements during encoding to gauge attention.

The effects of the musical manipulation demonstrate that a simple change in ambient audio can scale the experience of ambiguous threat. We unexpectedly found that a relatively neutral soundtrack, as compared with a horror film soundtrack, led participants to report stronger feelings of unpredictability. While the mechanism for this effect is unclear, it seems likely that the neutral soundtrack was at odds with the narrative and visual content of the world and, as a consequence, made the world more ambiguous. As others have observed (Roberts, 2014), when horror game soundtracks play continuously and do not change in response to events in the environment, players have no cues for risk or safety. The banal nature of the neutral soundtrack may have been particularly effective in eliciting this effect.

We also found that participants had a similar affective experience regardless of whether they controlled their navigation through the world. Although participants who watched another participant’s path through the world (instead of navigating themselves) found the world more interesting, they still experiencing it as creepy, unpredictable and threatening. So, although interactivity has a demonstrable link to presence within virtual environments (Cummings & Bailenson, 2016; Welch et al., 1996), these data suggest that a virtual world can nevertheless be emotionally evocative in its absence. Indeed, these data are in line with games research comparing players and spectators (Juvrud et al., 2021).

The paradigm presented here represents an integration of knowledge from disparate disciplines. In part, we relied upon empirical research into the nature of fear and anxiety. That body of research demonstrates that ambiguous threat emerges from temporal and spatial unpredictability. Environmental factors such as darkness, inescapability, the presence of hiding places, and the implied but undisclosed presence of hostile agents, have further been associated with feelings of ambiguous threat. We therefore included these features in the virtual world. But we also relied upon the expertise of video game design to bring these elements to life in a richly detailed, interactive environment. In recent decades, video games have exploded in popularity around the world, likely because of their ability to manipulate our emotions—i.e., to create feelings of excitement, fear, desire, and satisfaction. By employing game design strategies for emotional narrative building as well as common tropes from horror games, we built a world to evoke the feelings associated with ambiguous threat while examining concurrent cognitive and physiological responses.

One outcome of drawing inspiration from horror games and film is that some participants (e.g., fans of these genres) may have found the world more familiar than others. That familiarity may have, in turn, amplified or reduced their affective response. Indeed, prior experience of horror films predicts more positive responses to horror films, and “spoilers” can even amplify threat-related responses (Cantor et al., 1984; Johnson et al., 2020; Martin, 2019). With this in mind, future research could more closely examine the relationship between prior gaming or film-going experiences (or, indeed, with real life threat) and experiences in paradigms such as the Underwood Project.

Critically, the world is adaptable for future research. The virtual rooms are modular such that researchers can add, remove, or swap out rooms to change the length, shape, and nature of the experience. The content of each room is flexible so that researchers can include specific target stimuli or use the world to create stimuli (e.g., foils for a memory task). The world is also modular in terms of task goals. In the version of the world presented here, participants opened doors via a relatively simple search task. This task could be replaced to focus on, for example, decision-making or complex problem-solving.

The world is also adaptable in terms of platform. For the current study, participants experienced the world via desktop displays, but it is easily adaptable to use in immersive virtual reality via Unity assets (see kit for details). Given the immersive nature of VR and its ability to amplify arousal above and beyond desktop experiences (Kim et al., 2014), we expect that emotional experiences of experiencing this world in VR will meet and possibly exceed those reported here. Altogether, these features make the paradigm presented here useful for future research.

Although prior research demonstrates that ambiguous threat shapes affect, cognition, physiology, and behaviour (e.g., Cornwell et al., 2008; Grillon et al., 1991; Robinson et al., 2013; Walker et al., 2003), the manipulation of ambiguity in the laboratory has been relatively limited and we have more to learn about how these responses play out in complex, interactive environments such as the one presented here. By studying ambiguous threat in a context that evolves over time, we can better understand how the threat response itself unfolds, how individuals adapt to changes in context, and how differences in emotion regulation and cognitive performance emerge. By immersing participants in a three-dimensional environment, we can also examine attention and perception of both focal and peripheral stimuli and events. And by studying all of these things in an interactive environment, we can evaluate relevant behaviour under threat. Altogether, these benefits might help us better understand the challenges placed upon individuals who must live or work in hazardous environments and might, more generally, help us better understand how any of us might respond to the combination of threats and uncertainties that we must face, sooner or later, in everyday life.

### Supplementary Information

ESM 1 (DOCX 34 kb)
