# Supplementary material for: The underwood project: A virtual environment for eliciting ambiguous threat
Source: Behav Res Methods. 2022 Oct 26;55(8):4002–17. doi: 10.3758/s13428-022-02002-3 (PMC10700233; doi:10.3758/s13428-022-02002-3)
Supplement: Supplementary file 1 — (DOCX 34 kb) [file 13428_2022_2002_MOESM1_ESM.docx]

[https://osf.io/euvgt/?view_only=e88b28773d0f4fcab33cb8bae27fd862](about:blank)
